# The Resources and Requirements of the Voluntary Hospitals of London
*General Note.—The detailed figures of the 94 voluntary hospitals for each of the ten years covered by the tables may be found in Burdett's "Hospitals and Charities" for 1905


**Published:** 1905-02-11

**Authors:** Henry Burdett


### HOSPITAL ADMINISTRATION.

### CONSTRUCTION AND ECONOMICS.

### THE RESOURCES AND REQUIREMENTS OF THE VOLUNTARY HOSPITALS OF LONDON.*

By Sir Henry Burdett, K.C.B.

The following article is reprinted from the *Times*:—

THE HOSPITAL NEEDS OF LONDON'S POPULATION.

What are the hospital needs of London's population in the matter of free medical relief? It is, of course, impossible to answer this question directly. Indirectly, evidence accumulates every year to prove that the present system of free medical relief is wrong in principle, and in practice harmful to the self-respect and thrift instincts of the population. I have been in communication with the Registrar-General's office, and the figures I am about to quote, so far as population is concerned, are the most accurate and recent obtainable.

Taking the whole population of the county of London, and classifying it under the occupations of males and females aged 10 years and upwards on the Registrar-General's plan, I venture to attempt to indicate an answer to the question in the following way. I exclude from the classes who can claim to be entitled to free hospital relief the following:—

Civil Service officers and clerks; police; municipal, parish, and other local or county officers; officers, non-commissioned officers, and men in the Army and Navy; clergymen, ministers and priests, missionaries, Scripture readers, monks, nuns, and sisters of charity; barristers, solicitors, and law clerks; physicians, surgeons, dentists, and veterinary surgeons; school teachers, professors, and lecturers; authors, editors, journalists, and reporters; those engaged in scientific pursuits; civil and mining engineers; land, house, and ship surveyors; artists—*i.e.*, painters, engravers, and sculptors; architects; photographers; musicians, music masters, singers, and actors; merchants, brokers, agents, factors, salesmen, buyers, and commercial travellers; accountants; auctioneers, appraisers, valuers, and house agents; officers connected with commercial companies; bankers and bank officials and clerks; bill discounters, brokers, and finance agents; insurance officials, clerks, and agents; all persons retired from business, with pensioners and all who live on their means; and employers in all trades and businesses.

Of course this is necessarily an incomplete list, but the object is to show as far as possible what is the outside and excessive number of people who, in any circumstances, can persuade themselves to apply for free medical treatment to a London hospital. That the number I give is excessive will be admitted by everybody who examines the tables of occupation of residents in the county of London on page 76 *et seq.* of the census of the county of London. The actual number of possible hospital patients left in the census returns is 52 per cent. of the whole. The total population of the county of London in 1903 may be taken then at 4,613,812, of which 52 per cent., or 2,399,182 persons, with the exception of 75,558 paupers resident in institutions, may represent probable hospital patients. The net population under this category is therefore 2,323,624 people of all ages and both sexes, including some half million children under 10 years of age.

* General Note.—The detailed figures of the 94 voluntary hospitals for each of the ten years covered by the tables may be found in Burdett's "Hospitals and Charities" for 1905

Next let us consider the number of patients in London receiving free medical relief in 1903. They were:—

| | | |
|---|---|---|
| In-patients. | | |
| At voluntary hospitals... ... ... | 112,523 | |
| At infectious hospitals (mostly free cases) ... ... ... ... ... | 21,925 | |
| At Poor Law infirmaries (approximate) | 81,835 | |
| Total ... ... ... | | 216,283 |
| Out-patients. | | |
| At voluntary hospitals... ... ... | 1,646,976 | |
| At dispensaries (free) ... ... ... | 207,549 | |
| At dispensaries (provident) ... ... | 77,065 | |
| Poor Law dispensary and home cases | 122,129 | |
| Truss, &c., societies ... ... ... | 32,561 | |
| | | 2,086,280 |
| Grand Total ... ... | | 2,302,563 |

We thus find that the actual number of persons returned as receiving medical relief at institutions in the county of London in 1903 represents the whole number of possible hospital patients, with the exception of 20,000 persons, in round numbers. The increase in the population of the county of London in the 10 years 1894-1903—*i.e.*, that of 1903 compared with 1894—was 6 per cent., or 264,646 persons, and the increase in the voluntary hospital population alone during the same period was upwards of 23 per cent., or 330,466 patients, as the following table shows:—

*Beds and Patients Treated by the* 94 *Voluntary Hospitals in the County of London in the Years* 1894 *and* 1903.

| — | 1894. | 1903. | Percentage of Increase. |
|---|---|---|---|
| Beds ... ... | 9,110 | 9,771 | 7·25 |
| In-patients ... | 92,517 | 112,523 | 21·62 |
| Out-patients ... | 1,336,516 | 1,646,976 | 23 22 |
| Total Patients | 1,429,033 | 1,759,499 | 23·12 |

It will thus be seen that the voluntary hospitals admitted to treatment so many more patients in 1903 compared with ten years ago that the new patients exceed the whole additional population of London within the decade by 65,820—that is to say, whereas the population of London has increased by 6 per cent., the hospital population has increased by upwards of 23 per cent. No wonder that the general practitioners are everywhere complaining of the competition of the hospitals, and stating that they find it impossible to get a living in a community where free medical relief, in this excessive and ever-increasing volume, is given to practically all who apply for it. The hospital figures, too, may be taken to be worse than they look; for every year the best managed of the hospitals have exercised more care in returning the number of out-patients than was the case in 1894. It follows that the number of patients in 1894 is more likely to have been over-estimated than in 1903. Yet, although it was considered in 1894 that the hospital necessities of the Metropolis were more than met, the hospitals have voluntarily added to these obligations three times as much work at least as the natural increase of the population can have put upon them. This has been done, too, during the very period when heroic efforts were being made by the public to provide the hospitals with enough money to make them financially sound. In my view, every thoughtful person who studies these figures must be convinced that the hospital needs of London's population are at present more than provided for in the matter of free medical relief. There is a very real necessity for the enforcement of economy and control in regard to the admission of free patients to the London hospitals to-day. Out of this army of free patients a considerable proportion must be able to pay for most or all of the medical treatment they require. If this be so—and who that knows the facts can doubt it?—it follows that the hospitals must largely cut down the admission of free cases, by forcing the majority to obtain medical relief in the ordinary way. It is further incumbent upon the hospitals, in defence of their subscribers and in the best interests of the people of London of all classes, to bring the accommodation they afford to sick people up to the requirements of modern times by opening pay wards under conditions which will be adequate to protect and meet the reasonable claims of all the interests concerned.

### The Total Cost of the Work done by the London Voluntary Hospitals.

The money expended by the 94 voluntary hospitals in the county of London in 1894, on the maintenance of 9,110 beds and the treatment of 92,517 in-patients and 1,336,516 out-patients — that is, a total of 1,429,033 patients — was £837,725. At the end of the 10 years we find that these hospitals in 1903 expended no less than £1,341,641 on the maintenance of 9,771 beds and the treatment of 112,523 in-patients and 1,646,976 out-patients, making together a total of 1,759,499 patients treated in 1903. The following table gives the expenditure of the 94 hospitals during each of the 10 years, the total ordinary expenditure having increased from £703,635 in 1894 to £914,373 in 1903, and the extraordinary expenditure from £134,090 in 1894 to £427,268 in 1903. There was, then, an increase in the total expenditure under all heads in 1903, compared with 1894, of upwards of £500,000, or over 60 per cent.:—

*Expenditure of the Ninety-four Voluntary Hospitals in the County of London During the Ten Years*, 1894–1903.

| Year | Ordinary expenditure | Extraordinary expenditure | Totals |
|---|---|---|---|
| | £ | £ | £ |
| 1894 ... ... | 703,635 | 134,090 | 837,725 |
| 1895 ... ... | 705 246 | 144,401 | 849,647 |
| 1896 ... ... | 706,877 | 131 911 | 838,788 |
| 1897 ... ... | 743 617 | 112,724 | 856,341 |
| 1898 ... ... | 757,588 | 166,128 | 923,716 |
| 1899 ... ... | 761.940 | 228,812 | 990,752 |
| 1900 ... ... | 819,155 | 255,001 | 1,074,156 |
| 1901 ... ... | 864,523 | 247,680 | 1,112,203 |
| 1902 ... ... | 882,793 | 415,421 | 1,298,214 |
| 1903 ... ... | 914 373 | 427,268 | 1,341,641 |
| Totals for 10 years | 7,859,747 | 2,263,436 | 10,123,183 |

Perhaps the most remarkable feature of this table is the evidence it affords that, directly great efforts were put forward to finance the hospitals, the hospital expenditure went up by leaps and bounds. The Sunday Fund effort in 1895 synchronises with an increase of nearly £12,000, which disappeared in 1896. The King's Fund was established in February 1897, and the expenditure in 1898 shows an increase over that of 1896 of £85,000, or nearly 11 per cent. Thenceforward the growth is a steady one. Thus the increase over the previous year in 1899 was £67,000, in 1900 £83,000, in 1901 £38,000, in 1902 £186,000, and in 1903 £43,000. The total expenditure for the ten years was £10,123,183, of which £7,859,747 was ordinary expenditure—that is, money spent upon provisions, drugs, dressings, domestic expenses of all kinds, rent establishment and miscellaneous charges, salaries and wages, appeals and management generally—and £2,623,436 was extraordinary expenditure—*i.e.*, money laid out on permanent repairs, building improvements, and heavy items of this special kind. Finally, it should be noted that during the three years 1894–96 inclusive the ordinary expenditure of the hospitals did not, in fact, show any material increase; and the same may be said of the extraordinary expenditure. Considering all the foregoing facts and figures there is good evidence to show that the managers of the hospitals have the power, if they have the will, to control their expenditure year by year practically to any extent they may choose.

### Did the Public Provide the Money for the Increased Expenditure?

The public have nobly responded to the needs of the hospitals, as is shown by the following table, which gives the income of the 94 hospitals of the county of London during the ten years 1894 to 1903:—

*Income of the Ninety-four Voluntary Hospitals in the County of London during the Ten Years*, 1894-1903.

| Year. | Ordinary Income. | Legacies. | Special Donations for Rebuilding, etc. | Totals. |
|---|---|---|---|---|
| | £ | £ | £ | £ |
| 1894 ... ... | 619,362 | 133,580 | 54,843 | 807,785 |
| 1895 ... ... | 623,785 | 206,791 | 90,596 | 921,172 |
| 1896 ... ... | 608,362 | 117,342 | 53,261 | 778,965 |
| 1897 ... ... | 653,389 | 213,335 | 121,504 | 988,228 |
| 1898 ... ... | 656.017 | 233,637 | 158,368 | 1,048,022 |
| 1899 ... ... | 661,295 | 275,960 | 107,026 | 1,044,281 |
| 1900 ... ... | 673,203 | 197,272 | 106,725 | 977,200 |
| 1901 ... ... | 712,360 | 299,022 | 227,042 | 1,238,424 |
| 1902 ... ... | 758,722 | 334,479 | 201,476 | 1,294,677 |
| 1903 ... ... | 741,557 | 283,494 | 306,039 | 1,331,090 |
| Totals for ten years | 6,708,052 | 2,294,912 | 1.426,880 | 10,429,844* |

* Exclusive of the King's Hospital Fund capital—say, £800,000.

The above income figures include, of course, all the dividends and receipts from invested property possessed by the 94 hospitals.

The lean year was 1896; but, with the exception of that year and 1894, when the sum received was £30,000 less than the total expenditure, the contributions of the public to the hospitals, when legacies are included, have practically more than sufficed to meet the expenditure each year. For, although in 1900, the receipts were £97,000 short of the expenditure, in 1901 they exceeded the total expenditure by

£126,000. This shortage in 1900 was due to the fact that in that year the yield from legacies was relatively small. Yet the yield from legacies has steadily increased, and may now be considered as trustworthy an item of revenue as any other source of voluntary contributions. In 1902 the hospitals received £334,479 from legacies alone, and the average and actual contributions under this head during the last three years of the decade show an increase of upwards of 100 per cent. over the receipts in 1894.

The total receipts for the ten years, £10,429,844, exceeded the total expenditure, £10,123,183, of the 94 hospitals by £306,661. It will thus be seen that the 94 hospitals received collectively, but directly, an average of £30,600 a year more than they expended year by year—a fact which we hope will dispose once and for all of the wild statements that the voluntary system of support has broken down, and that the financial position is so grave as to call for rate support or Governmental aid.

### Capital Expenditure and Reserves.

It will be noticed that the extraordinary or capital expenditure of the 94 voluntary hospitals during the ten years has amounted to upwards of 2¼ millions. The appeals for huge sums which this expenditure has entailed have made the public and the Press not unnaturally apprehensive at the financial outlook of the London hospitals. This apprehension is deepened by the knowledge that reconstruction and new building works, to the value of £1,000,000 in round figures, have still to be provided for. In my view these apprehensions are not well founded, for directly the works in question are completed and paid for there should be practically an end to capital expenditure of this kind for many years to come. The history of our hospitals shows that great calls have been successfully made for large sums of money for hospital buildings in the past, and that, owing to an awakening of public opinion, such appeals have usually come almost together. If the hospital authorities realise that, when the present building operations are completed, they must organise their finances so as to add gradually to their capital year by year, the reserves which the completion of the building operations will place in their hands should prove more than ample for this purpose.

First, then, the hospitals have behind them, apart from sites, invested funds which cannot be less than £4,000,000 in value; next, the reserve represented by the invested capital of the King's Fund, say £800,000; next, a further reserve which will become available directly the rebuilding and reconstruction works now in progress have been finally completed and paid for. The extraordinary expenditure for these purposes during the last 10 years has amounted to £2,263,436, and a careful estimate of the cost of the additional extensions, including the rebuilding of St. George's Hospital, the completion of the new King's College Hospital, the works still in progress at the London, Guy's, and other hospitals, and the requirements of a number of smaller institutions, amounts to about £1,000,000 sterling.

Fortunately these appeals for rebuilding have been delayed until the means for raising the revenue by each individual hospital and through the central funds have been so organised as to provide each year the necessary funds for maintaining the hospitals with the maximum of efficiency, with the exception of about £100,000. It follows that, as the appeals on account of capital for buildings are temporary and special, the future financial outlook of the 94 hospitals within the county of London supported by voluntary contributions should be secure and satisfactory.

### The Enforcement of Economy.

The one paramount necessity of the moment is the enforcement of economy in regard to admissions and the expenditure of our hospitals in London and elsewhere. The great changes brought about by Lord Lister's discoveries and the introduction of trained nurses and modern methods of treatment have led the authorities into large expenditure, which in the first stages of these developments, owing to a natural desire not to cripple efficiency, has been too prodigal in many directions. The same absence of effective control characterises the methods of admission to the in- and out-patient departments of our hospitals. At the present time the hospitals are expending more money than they need for the maintenance of the highest efficiency through (1) an absence of effective control over expenditure, and (2) the admission of a host of patients, especially in the out-patient department, who ought never to be admitted to free hospital treatment at all. These defects must be remedied, for then the present contributions to the London hospitals can be made adequate to pay for all the work that they can properly and usefully undertake.

### A Word of Caution.

For upwards of 30 years I have been actively working to promote the efficiency and to secure adequate financial support under the voluntary system for the hospitals of this country. It is the fact that the large majority of the population still give little or nothing to the hospitals, and that to a great extent the increased contributions have come from the few, rather than the many, who have generously aided the cause of the sick from the first. But I am grateful for the knowledge that, thanks to the King's Fund and the League of Mercy especially, an increasing number of people are each year attracted to give money to the voluntary hospitals.

The facts given in this article mark an epoch in the history of the voluntary hospitals of London. The figures will afford an interesting basis for future comparisons; for the hospital managers are fully alive to the importance of economy, and anxious to secure it. Within the last few days I have been approached by some of the managers of the largest and most important enterprises, responsible for the purchase in the best markets and for the supply of goods of all kinds to the public, who are prepared to place the machinery and organisation of their large businesses and the services of their staff at the disposal of the hospitals in an absolutely honorary capacity.

It is a good sign, too, as my correspondence shows, that hospital managers not only in London, but throughout the country, have become warmly interested in the economical considerations which underlie the figures and facts I have given in this article. All these considerations should give the public confidence, and win their support in increasing measure to the voluntary hospitals, and to the great central funds which are doing so much to promote economy and secure the permanency of the voluntary system in this country.

---

A sufficient sum of money has been raised for the purchase of the Crown land near the Combermere Barracks for the erection of the new Windsor and Eton Royal Infirmary. The King and Queen and other members of the Royal Family have contributed to the fund.

---

Among the latest contributions received at the Bank of England for King Edward's Hospital Fund for London are the following:—Annual subscription, the Bank of England, £250; Messrs. Chalmers, Guthrie, and Co., Limited, £25; Mr. Henry Oppenheim, £25. Donations—Mr. Henry Oppenheim, £26 5s.; employés of Messrs. Mabie, Todd, and Bard, £16 10s.

